# *Lactobacillus casei* extracellular vesicles stimulate EGFR pathway likely due to the presence of proteins P40 and P75 bound to their surface

**DOI:** 10.1038/s41598-020-75930-9

**Published:** 2020-11-06

**Authors:** Christine Bäuerl, José M. Coll-Marqués, Carmen Tarazona-González, Gaspar Pérez-Martínez

**Affiliations:** Laboratory of Lactic Acid Bacteria and Probiotics, Department of Food Biotechnology, Instituto de Agroquímica y Tecnología de Alimentos, Consejo Superior de Investigaciones Científicas (CSIC) (Spanish National Research Council), Avenida Agustín Escardino, 7, 46980 Paterna, Valencia, Spain

**Keywords:** Bacteria, Bacterial host response

## Abstract

In the complex interplay of beneficial bacteria with the host, there are few examples of bacterial metabolites and effector molecules that have been consistently identified. Protective effects on the intestinal epithelium have been ascribed to P40 and P75, two well characterized cell wall muramidases, present in the culture supernatant of strains belonging to the taxon *Lactobacillus casei/paracasei/rhamnosus*. This work reports that *Lactobacillus casei* BL23 extracellular vesicles (BL23 EVs) have a small size (17–20 nm or 24–32 nm, depending on the method used) and contain lipoteichoic acid (LTA). Interestingly, all detected P40 and most of P75 were associated to EVs and possibly located at their external surface, as shown by proteinase K digestion. Biosensor assays showed that both proteins bind LTA and vesicles, suggesting that they could bind to ligands like LTA present on BL23 EVs. Native BL23 EVs have a moderate proinflammatory effect and they were able to induce phosphorylation of the epidermal growth factor receptor (EGFR), showing an effect similar to purified P40 and P75 and leading to the conclusion that the activity described in the supernatant (postbiotic) of these bacteria would be mainly due to P40 and P75 bound to EVs.

## Introduction

EVs are produced by all domains of life, including eukaryotes, bacteria, parasites, fungi and archae. In Gram-negative, generation of EVs has been described as a “pinch off” process from the outer membrane and hence they were referred to as outer membrane vesicles (OMVs). They contain cargoes of very different nature, like virulence factors, adhesins, DNA, RNA, communication compounds, immunomodulatory factors or toxins^1–3^. A great amount of the current knowledge on bacterial microvesicles come from such OMVs due to their implication in virulence processes. For some time, it was believed that Gram-positive bacteria were not able to secrete extracellular vesicles due to their thick cell wall^4^, but Gram-positive pathogenic species, such as *Staphylococcus aureus, Bacillus anthracis* or Group B *Streptococcus*, produce membrane-derived vesicles and some of these species EVs induce inflammatory effects similarly to the complete bacteria^5–7^. For example, toxin components of the anthrax toxin of *B. anthracis* were associated to EVs inducing a robust immune response^5^. Further, clinically relevant isolates of *Enterococcus faecium* produce EVs containing antibiotic resistance related proteins and virulence factors that may aggravate its virulence^8^. Also, EVs from probiotic bacteria have comparable effects to whole bacteria, increasing the number of T_reg_ cells, in case of *L. rhamnosus* JB-1^9^, and promoting the survival of *Caenorhabditis elegans* exposed to vancomycin-resistant *E. faecium* by *L. plantarum* WCFS1^10^. Analysis of EVs from probiotic lactobacilli, like *Lactobacillus rhamnosus*, *L. plantarum*, *L. reuteri* and *L. casei*, showed that they contained glycolytic enzymes, chaperonins^9,11–14^ and ligase functions^15^. In addition to proteins and membrane phospholipids, EVs from Gram-positive bacteria also contain lipoteichoic acid (LTA). LTA is an anionic glycopolymer—normally glycerolphosphate—present in Gram-positive bacteria^16–18^ and, of course, also in *Lactobacillus*^19–21^. LTA’s diacylglycerol residues are embedded in the phospholipid bilayer, exposing the LTA negatively charged glycerolphosphate at the outer surface of the bacterial membranes, negative charges that are modulated mainly by D-alanylation. LTA together with wall teichoic acid (WTA) play an essential role stabilizing the cell wall by covalently binding to N-acetyl-muramic acid of the peptidoglycan and providing the net negative charge of the bacterial surface. They are very important for biofilm formation, a target of antimicrobial agents and powerful surface antigens^16,17^.

*Lactobacillus casei* BL23 (BL23) is a well-studied probiotic strain for which anti-inflammatory and anti-tumor effect have been described in mouse models^22–25^. Two secreted cell wall muramidases that contain a CHAP (NlpC/P60) domain have been characterized in BL23, P40 and P75, and homologs to these proteins are exclusively present in the *L. casei* taxon, that comprises mainly the species *L. casei, L. paracasei and L. rhamnosus*^26^. Their ability to stimulate growth and survival of epithelial cells was first described in *L. rhamnosus* GG^27^. These proteins proved to have anti-apoptotic and anti-inflammatory effects mediated by the activation of the EGFR/Akt pathway. Then, it was shown that protein P40 reduces dextran sulfate sodium (DSS)-induced experimental colitis, and it induced mucin synthesis by intestinal epithelial cells (IEC), intestinal IgA production and promoted intestinal development in early life^26,28–31^. The abnormal cellular morphology of knock out mutants of *L. casei* and *L. rhamnosus* lacking these proteins showed that they are involved in the normal conformation of the bacterial cell wall; P75 is a γ-D-glutamyl-L-lysyl-endopeptidase required for daughter cell separation^28,32^.

This work was first motivated by the fact that both, P40 and P75, have secretory signals, they were processed and found in the culture medium but, intriguingly, they had also been described as part of the cargo of *L. casei* BL23 EVs after proteomic analysis^13^. Here we have managed to explain, first how P40 and P75 can be secreted but also associated to EVs, and also that *L. casei* BL23 derived EVs have an equivalent activity to P40 and P75, specifically on the EGFR/Akt pathway in IEC.

## Results

### Isolation of *L. casei* BL23 EVs (BL23 EVs) and detection of proteins P40 and P75 on BL23 EVs

**Figure 1:**
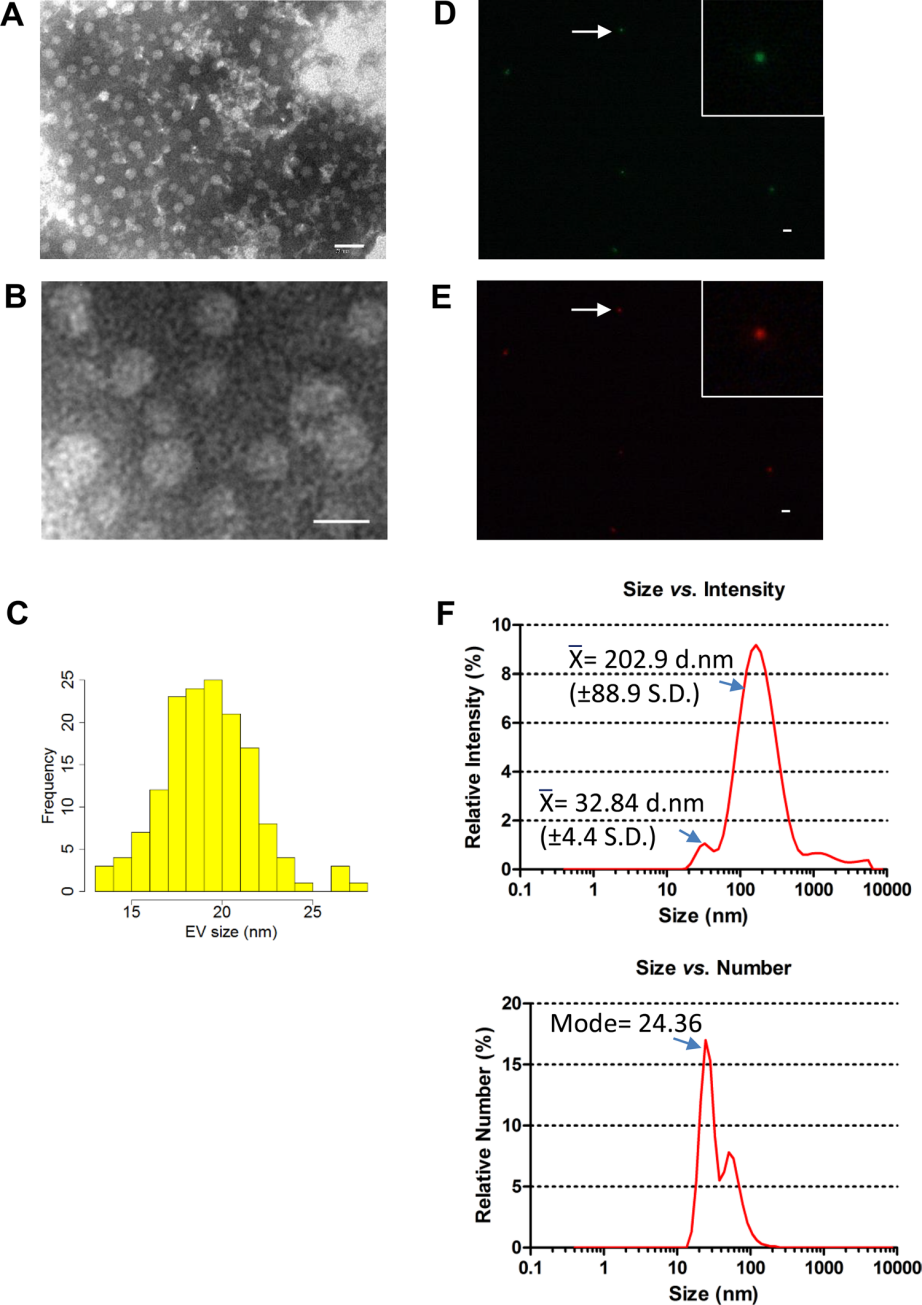
*Lactobacillus casei* BL23 releases membrane vesicles. (**A**, **B**) Representative TEM images from BL23 EVs. Scale bars: 50 nm for image (**A**) and 20 nm in (**B**). (**C**) Size-frequency distribution representation as determined based on TEM. (**D**) Fluorescence images of EVs derived from *L. casei* BL23 [pT1-GR::p127] expressing *gfp* or (**E**) *rfp*. Scale bar: 2 µm. (**F**) DLS determination of the EV sizes represented as Size distribution by Intensity or by Relative Number.

BL23 EVs were isolated by ultracentrifugation from culture supernatant, filtered through 0.45 µm microbiological filter, and they were observed by transmission electron microscopy (TEM) negative staining (Fig. 1A,B). Electron micrographs showed EVs with a relatively uniform spherical-like shape, which had a narrow size distribution ranging from 13 to 28 nm of diameter, with mean of 19.21 nm (+ / − 2.51 nm S.D.) (Fig. 1C). This size is similar to previously described *L. casei* EVs^19^. EVs from *L. casei* BL23 [pT1-GR::p127], expressing both the genes of red (*rfp*) and green fluorescent proteins (*gfp*), were isolated and visualized by fluorescence microscopy together with BL23 EVs as a control for autofluorescence. We could detect in recombinant BL23 EVs both green and red fluorescence (Fig. 1D,E), indicating the presence of these fluorescent proteins in EVs which in the parental cell are localized in the cytosolic compartment. Size of BL23 EVs was also estimated by Dynamic Light Scattering (DLS) and good quality data were obtained for vesicles isolated by ultracentrifugation (Fig. 1F). Average size *vs.* intensity estimation showed one large peak of 202.9 nm (+ / − 88.9 nm S.D.) diameter, but since larger particles exponentially scatter more light intensity^33^ the majority of vesicles corresponded to the smaller intensity peak of 32.84 nm (+ / − 4.4 nm S.D.) diameter, as confirmed by determination of size *vs.* relative number (mode = 24.36 nm diameter) (Fig. 1F). These data are in agreement with the measurements obtained by TEM and explain the absence of larger particles using this method.

**Figure 2:**
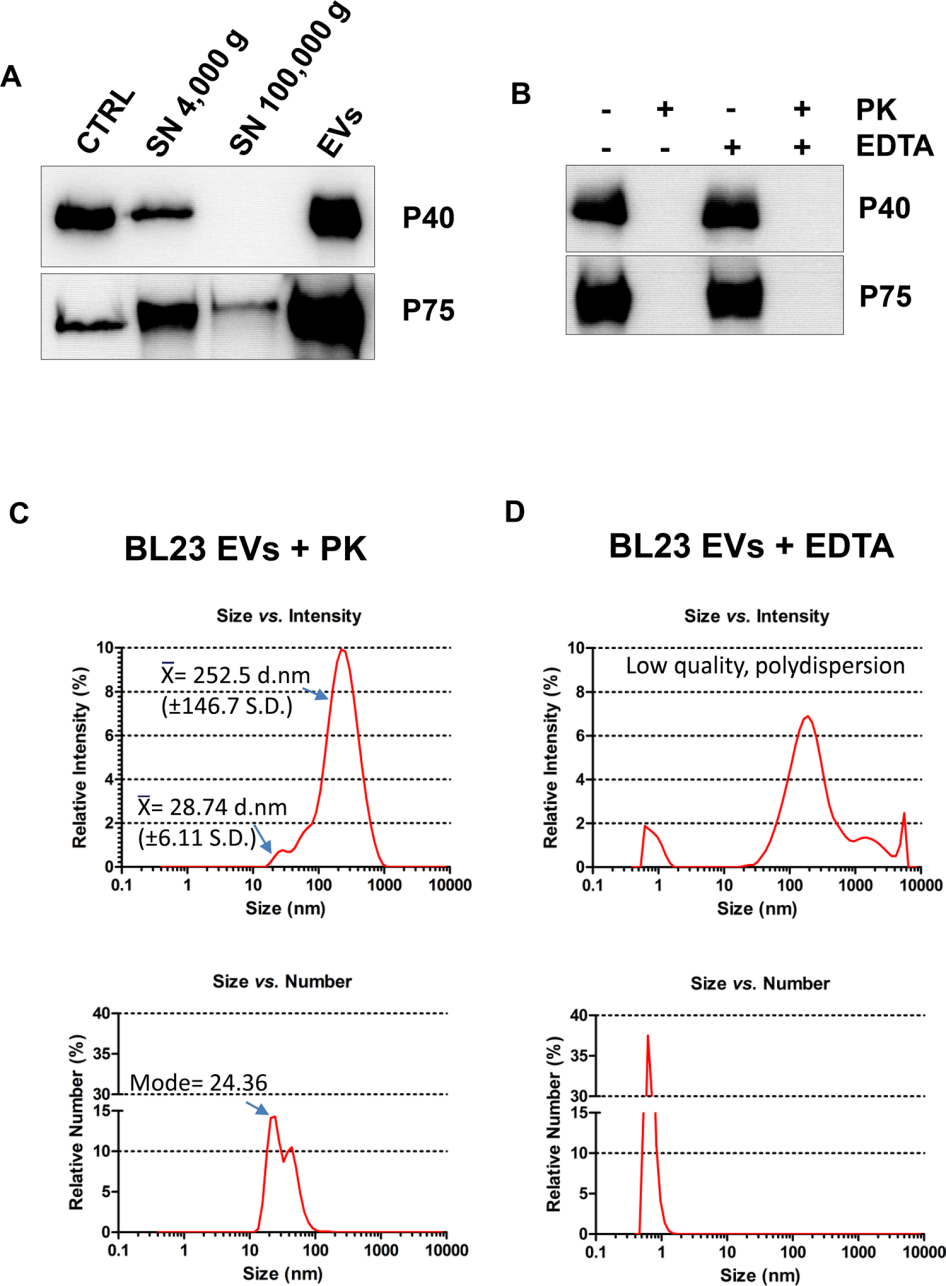
P40 and P75 are abundant in extracellular EVs. (**A**) Western-Blot detection of P40 and P75 in different fractions: (CTRL) control lanes contain 10 ng of purified recombinant His-tagged P40 or P75, (SN) were loaded with 15 µl of supernatant resulting from centrifugations at 4000* g* and 100,000* g*, (EVs) contain EVs quantified as 1 µg of total protein. (**B**) Western-blot analyses of EVs treated ( +) or untreated ( −) with proteinase K (PK) or with 0.1 M EDTA (EDTA). (**C**) DLS determination of sizes in samples treated with proteinase K (PK) or with EDTA.

The presence of multifunctional proteins P40 and P75 in isolated EVs was determined by western blot (Fig. 2A) using specific antibodies against their respective N-terminal domains. When band intensities were compared with the control lanes, with 10 ng of the respective purified proteins, sample amounts of 19.7 ng (+ / − 9 ng S.D.) for P40 and 73.6 ng (+ / − 12.1 ng S.D.) for P75 could be inferred. This represents 1.9% (+ / − 0.9% S.D.) and 7.4% (+ / − 1.2% S.D.) of P40 and P75, respectively, of the total EV protein load, suggesting that they have—particularly P75—a very relevant contribution to the EV protein composition. The presence of P40 and P75 was monitored in each step of the preparation of EVs. Both were detected in the initial culture medium supernatant (SN 4,000 ×*g*), but after the ultracentrifugation (UC) step (100,000 ×*g*) no soluble P40 could be found in the supernatant, whereas a small fraction of P75 was still present in the supernatant. To investigate the localization of both proteins in the EVs, EVs were subjected to proteinase K assay^34^ and EDTA treatment, where proteinase K would degrade all surface exposed proteins of intact vesicles, while in EDTA-lysed EVs all proteins would be accessible to proteinase K digestion. As shown in Fig. 2B, both P40 and P75 were completely digested by proteinase K with and without EDTA, suggesting that they are localized at the external surface of EVs. In order to determine whether proteinase K would affect the stability of EVs, BL23 EVs treated separately with proteinase K and EDTA were examined by DLS. Size distribution of proteinase K treated vesicles (Fig. 2C) was comparable to intact BL23 EVs, while EDTA treatment rendered a remarkable DLS polydispersion, high intensity and high counts of very small size particles (debris) (Fig. 2D), that were falling below the sensitivity of the equipment (1 nm). As consequence, quality parameters in the determination of particle size indicated “low quality data”. This would be in agreement with the destruction of the vesicles.

### P40 and P75 have affinity for LTA and vesicles

**Figure 3:**
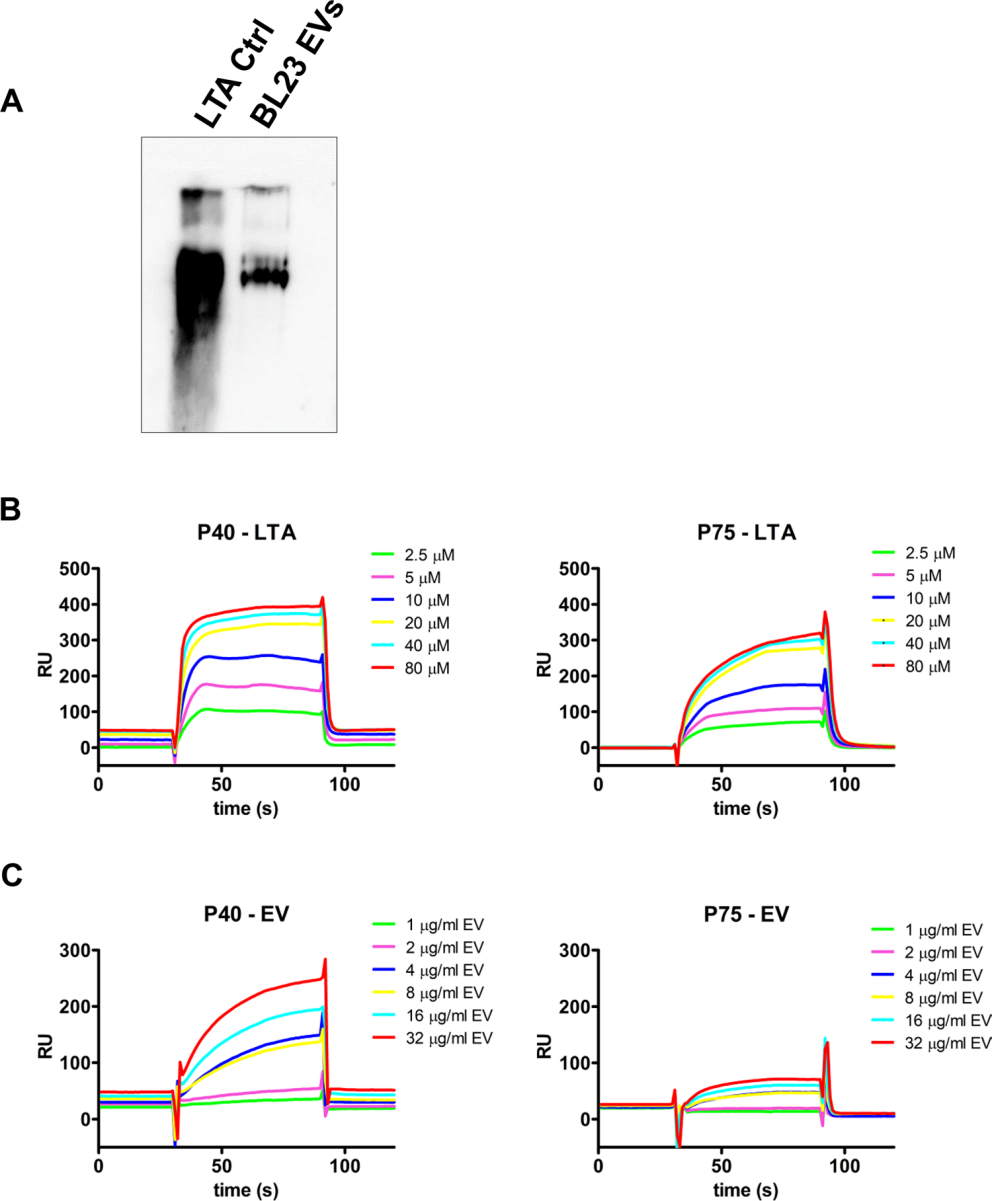
LTA on vesicles and surface plasmon resonance binding assays of LTA and BL23 EVs to P40 and P75. (**A**) Western blot from a non-denaturing 17.5% PAGE loaded with LTA (Control) and *L. casei* BL23 EVs. (**B**) sensorgrams showing arbitrary resonance units of LTA to immobilized P40 and P75, and (**C**) sensorgrams of BL23 EVs binding to P40 and P75.

As shown by the proteinase K assay, P40 and P75 are distributed at the surface of the EV. Teichoic acids—LTA and WTA—are essential elements of the bacterial surface in Gram-positives. LTA are anchored to membranes of *L. casei* EVs^19^, so it was interesting to test the possible affinity of P40 and P75 to LTA. First it was necessary to confirm the presence of LTA in BL23 EVs, but detection of LTA required to use greater concentrations of EVs (2.85 μg of EVs protein per sample) than the detection of P40 and P75, so, EVs were prepared with the PEG6000 procedure^35^ (Fig. S5). LTA in BL23 EVs could be directly detected by western blot after non-denaturing 17.5% polyacrylamide gel electrophoresis (PAGE) (Fig. 3A). This method was used for LTA quantification (Fig. S7), and the amount of LTA relative to protein in *L. casei* Bl23 EVs was 0.059, that corresponds to a mass ratio of 1:16.9 (LTA:protein). The precise molar ratio of LTA to total EVs protein—and to P40 and P75—could not be determined because we lacked accurate molecular size estimations. Nonetheless, considering that the estimated proportion of P40 and P75 was 1.9% and 7.4% of the EVs protein, respectively, and that average LTA weight would be much smaller^36^ than the average protein mass in EVs^13,14^, we estimated that there would be sufficient LTA on the EVs to bind both P40 and P75.

Then, in order to prove that P40 and P75 interact with LTA, a SPR biosensor assay was designed in which both proteins were immobilized on CM5 chips and LTA were circulated in the microfluidic cells. As shown by the sensorgrams, LTA had great binding affinity for P40 and P75 (Fig. 3B). The inherent variability of the molecular size of LTA and the formation of variable size micelles in water^37^ were the possible cause of low matching with available kinetic models. Nevertheless, testing increasing LTA concentrations showed SPR sensorgrams with a clear concentration dependent response (Fig. 3B, Fig. S8A and S8B), from which tentative association (K_A_) and desorption (K_D_) constants were calculated and suggested a higher affinity with slower desorption of LTA for P40 than P75 (Supplementary Data, Table S1). The possible attachment of both proteins to the external LTA on *L. casei* BL23 EVs was confirmed by testing the binding capability of whole EV to both proteins in the biosensor chip (Fig. 3C), and also that the SPR signal was concentration dependent (Fig. S8B). The higher affinity of P40 to both LTA and EVs would explain that all P40 produced by BL23 during growth would remain bound to EV, but some P75 could be unbound in solution. Therefore, the presence of LTA in BL23 EVs and the interaction of P40 and P75 with LTA molecules has been confirmed, although surface binding to other elements cannot be discarded.

### Effect of EVs on intestinal epithelial cells (IEC)

**Figure 4:**
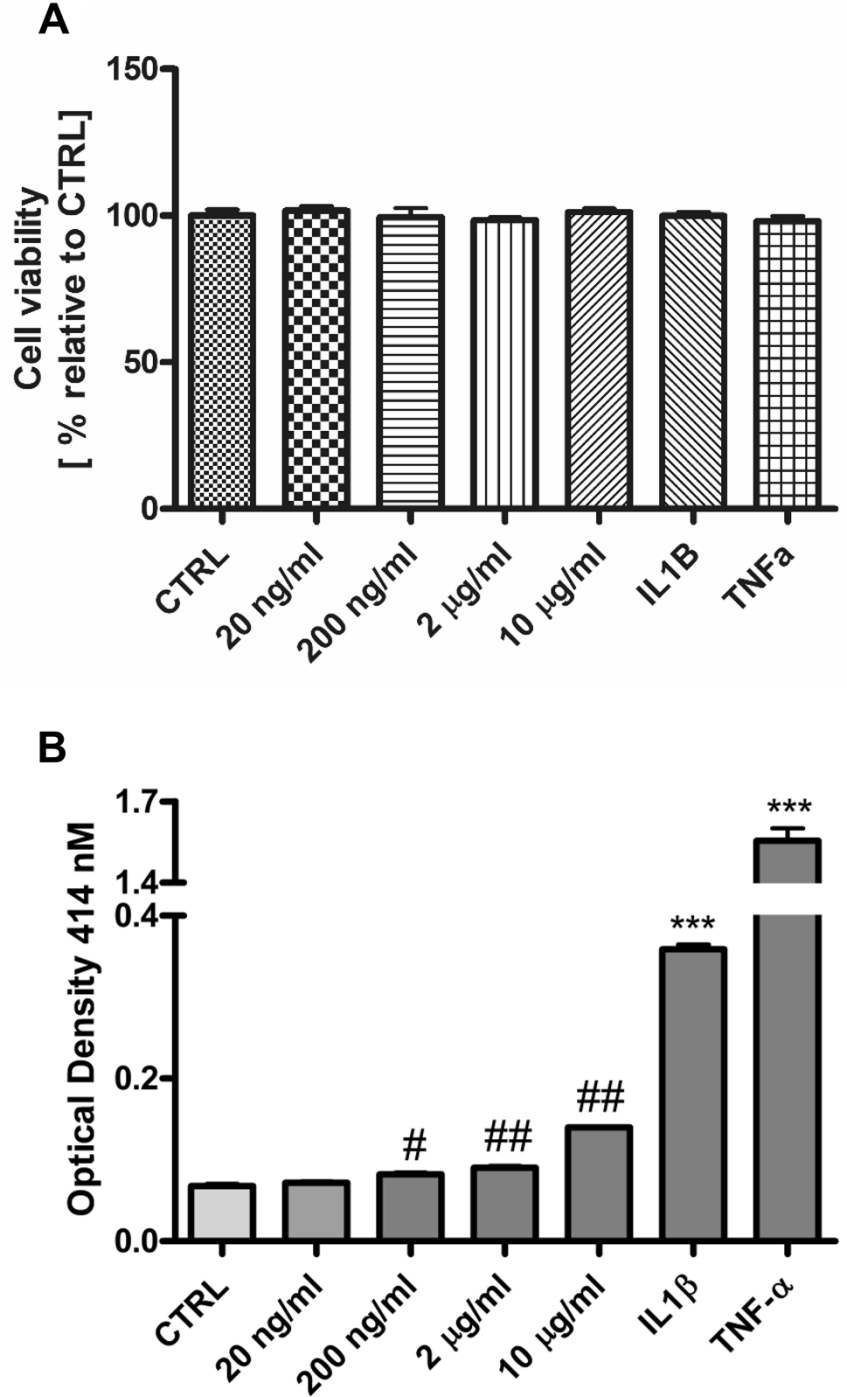
Response of epithelial cell cultures to the presence of purified BL23 EVs. (**A**) Cell viability measured using the resazurin reduction assay after incubation of HT-29 #16 cells with different concentrations of BL23 EVs (20 ng/ml to 10 µg/ml), IL1-β and TNF-α. (**B**) HT-29 #16 cells bearing phosphatase activity under the control of NF-kB were treated for 24 h with different concentrations of BL23 EVs (20 ng/ml to 10 µg/ml) and IL-1β and TNF-α (both 10 ng/ml). Cultured cells were collected and the reporter gene for secreted alkaline phosphatase activity was measured. Statistical analysis was performed using one-way ANOVA test, ***p < 0.001 compared to Control using Dunnett’s post test and student’s t-test, ^#^*p* < 0.05, ^##^ < 0.001 compared to Control.

First, it was essential to check that BL23 EVs did not affect the viability of IEC by a simple and sensitive viability assay with resazurin. Indeed, there was no significant effect on viability of any of the concentrations tested (Fig. 4A). Then, the pro- or anti-inflammatory potential of BL23 EVs was evaluated using a specific IEC NF-kB reporter assay in stably transfected HT-29 cells that expressed secreted alkaline phosphatase under the control of the transcription factor NF-kB. NF-kB-driven gene-expression was induced with the pro-inflammatory cytokines TNF-α and IL-1β and EVs did not reduce inflammatory signals (Fig. S9). The pro-inflammatory effect of EVs was assayed noticing a very moderate twofold increase in the expression of the NF-kB dependent promoter with respect to Control (OD_414_ 0.0677) only at the highest concentration assayed (10 µg/ml, OD_414nm_ 0.1397), which compared to the proinflammatory stimulants IL-1β (OD_414nm_ 0.3590) and TNF-α (OD_414nm_ 1.554) represents a very low response (Fig. 4B). These results may be due to the complex composition of EV with surface LTA and other possible cargoes.

**Figure 5:**
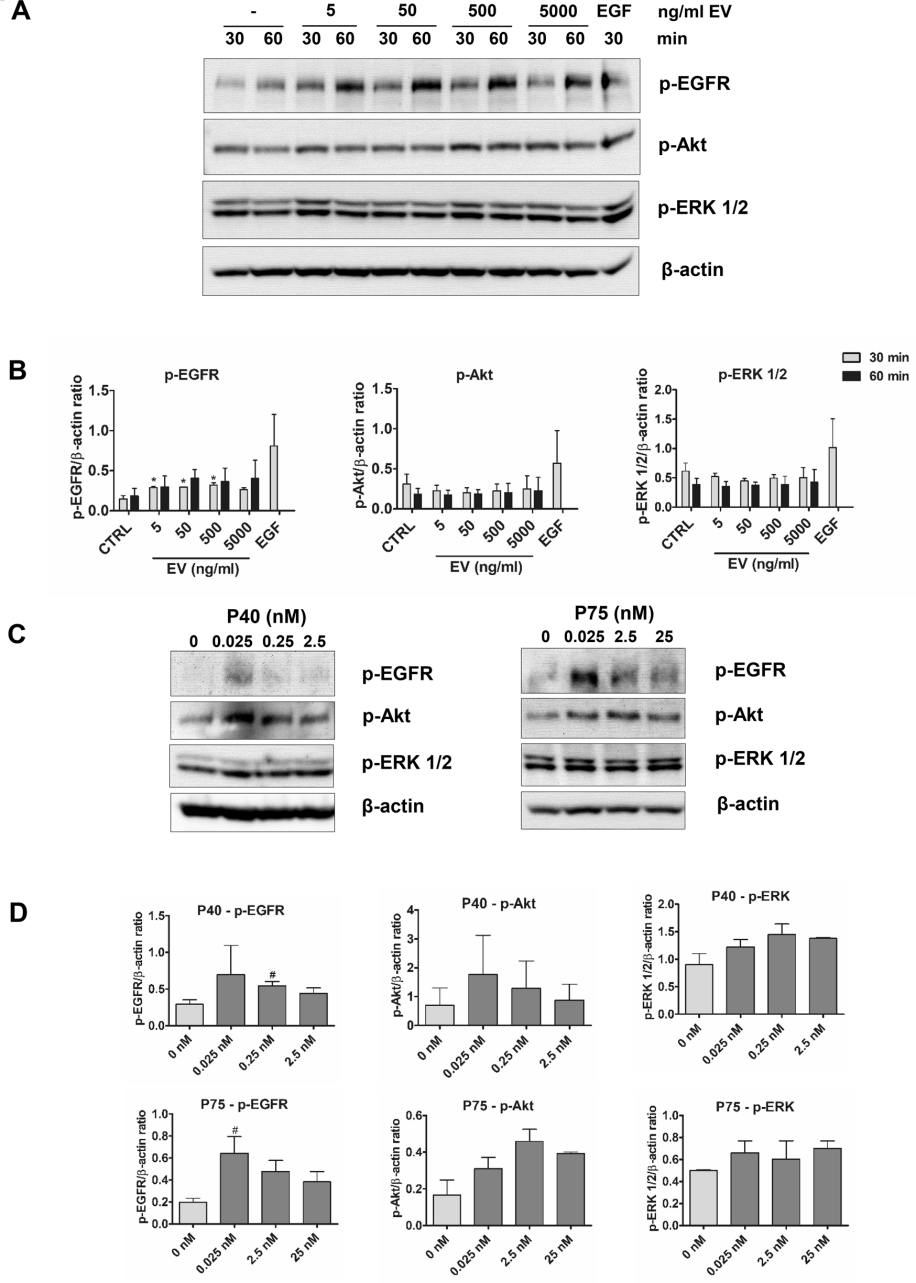
Activation of p-EGFR in T84 IEC in response to EVs. (**A**) Expression of p-EGFR, p-Akt and p-ERK 1/2 in response to BL23 EVs and epidermal growth factor (EGF). (**B**) Graphs represent the quantification of p-EGFR, p-Akt and p-ERK1/2. Statistical analysis was performed using one-way ANOVA followed by a Dunnett’s post test compared to the corresponding Controls at 30 min and 60 min, *p < 0.05. (**C**) Expression of p-EGFR, p-Akt and p-ERK 1/2 in response to purified P40 and P75 when stimulated during 1 h. (**D**) Graphs represent the quantification of p-EGFR, p-Akt and p-ERK1/2. Statistical analysis was performed using student’s t-test, ^#^*p* < 0.05 compared to Control.

Both, P40 and P75 proteins, were described to have anti-apoptotic properties inducing the phosphorylation of EGFR and, in the case of P40, to protect the intestinal epithelium from induced inflammation in an animal model^28,29^. Since those proteins have been found bound to BL23 EVs, it was interesting to assay EGFR activation by BL23 EVs in line T84 of IECs (Fig. 5A). Interestingly, BL23 EVs could activate the phosphorylation of EGFR in a dose-dependent manner (5–5,000 ng/ml of EV protein) (Fig. 5A,B). No activation of other signal transduction pathway intermediates could be observed (p-Akt, p-ERK1/2), in fact, basal levels of phosphorylated Akt and ERK1/2 were reduced after treatment with BL23 EVs. The efficient activation of EGFR/Akt intermediates by purified P40 and P75 proteins is also shown as reference (Fig. 5C,D). A biphasic effect (saturation of stimulus), or hormesis, was noticeable in the assays using BL23 EVs and P40 and P75.

## Discussion

The formation of EVs is a characteristic of all three domains of life and EVs have received increasing attention as mediators of intercellular communication via transfer of a wide variety of molecular cargoes. The particular role of bacteria-derived membrane vesicles in the interplay between the host and the microbiota, particularly the gut microbiota, is only starting to be deciphered. EVs are shed into the intestinal lumen, there they can act in distance to their parent cells^38^ and they are able to cross the mucus layer, thereby getting in direct contact with the intestinal epithelium. Moreover, they are resistant to enzyme degradation and low pH, and recent studies suggest that EVs can possibly cross the intestinal epithelial barrier, reach the bloodstream and spread to peripheral tissues^39,40^. EVs from Gram-positive and Gram-negative bacteria have a different origin. In *E. coli* they are predominantly derived from the outer membrane^15^, while the generation and release of EVs from Gram-positive bacteria is still not fully elucidated, but first evidences point to the involvement of cell wall-degrading enzymes that are able to weaken the cell wall and to release EVs^2,41,42^.

Historically, a number of functional proteins have been found in culture supernatants of probiotic lactobacilli and bifidobacteria that were lacking secretion signal sequences^43,44^. Recent proteomic studies of EVs isolated from culture supernatants of probiotic bacteria have shown that they are present in EVs, as in the case of *L. casei* BL23^13^ and *L. rhamnosus*^9^. EVs from the probiotic strain *E. coli* Nissle 1917 showed protective effects on IEC cultures^45^, but there are very few reports on the functional properties of EVs from lactobacilli^9,10^ and none on their mechanism of action.

In the past, P40 and P75 production by *L. rhamnosus* and *L. casei* BL23 has been tested in the culture supernatants assuming they were secreted proteins^26,28,46^, but the first proteomic studies of *L. casei* EVs found both functional proteins associated to EVs^13,14^. In fact, a considerable amount of P75 detected was associated to EVs (7.4 % +/− 1.2 % S.D. of total detected protein), in agreement with previous proteome analysis of *L. casei* ATCC393 EVs. This study disclosed by immunological methods that in fact, under standard experimental conditions, all secreted P40 and the major part of P75 were associated to EVs and demonstrated that EVs are able to reproduce the functionality of the purified proteins, determined as EGFR activation. It can be inferred that EVs are mediating the immunomodulatory properties ascribed to supernatants or conditioned medium from *L. casei*^47^ and by extension possibly of the *L. casei/paracasei /rhamnosus* group^26,48^ and this can be most relevant in studies, where bacterial culture supernatants or conditioned medium would be -or have been- used to study *in vitro* or *in vivo* responses in human or animal models.

Here, proteinase K digestion assays showed that both proteins are located at the exterior of EVs. LTA and wall teichoic acid (WTA) are anionic glycopolymers covalently attached to peptidoglycan in Gram-positive bacteria and with a high affinity for cations^16,49,50^. SPR biosensor assays definitely confirmed that LTA have high affinity for P40 and P75 and sensorgrams and calculated absorption and desorption constants indicate that P40 had greater affinity. Therefore, it could be inferred that P40 and P75 are externally bound to EVs by interaction with LTA, possibly by attractions between charged or polar amino acids and the negative charges of LTA. The corresponding SPR assays showed that BL23 EVs clearly interacted with P40 and P75 and, although these results cannot discard other forms of interaction with EVs, they confirm their likely surface attachment to EVs. LTA displayed a lower binding ability to P75, a difference with P40 that was more remarkable in case of EVs and that could be explained by the synergy of the lower affinity and a likely saturation of LTA with other bound molecules. As mentioned above, teichoic acids have great affinity for cations and treatments with high concentrations of LiCl have been used for long time to release possible ligands from LTA and WTA^51,52^. In fact, cell wall associated proteins are efficiently extracted from bacterial cultures with high concentrations of LiCl, for example P40 and P75^28^. This information together with data obtained here suggest that both proteins are likely bound to LTA and WTA in the bacterial cell wall^20^.

Additionally, EVs may have a renewed interest for the use and the detailed characterization of other proteins and effectors found in the culture supernatant of probiotic bacteria^53^. In fact, since EVs are not living organisms, they could facilitate production, legal and public acceptance and could help in the scientific substantiation of their benefits. These facts can have a great relevance in the emerging field of postbiotics^54^. Further, from the biotechnological stand point bacterial EVs also show a great potential as vehicles for vaccines and therapeutics^55,56^, as they are also able to be internalized in eukaryotic cells^34,57^ and cross the epithelial barrier^58^.

In conclusion, this work showed that the two functional proteins P40 and P75 are bound at the surface of BL23 EVs, possibly attached to LTA, and confirmed that EVs activation of EGFR is similar to recombinant P40 and P75 purified proteins^28,29^, which constitutes the first report describing that EVs from probiotic bacteria contain bacterial proteins responsible for their in vitro activity. These findings highlight the importance of EVs in the complex communication network between the host and beneficial bacteria, and possibly other members of the gut microbiome.

## Material and methods

### Bacterial culture and isolation of extracellular vesicles

EVs were prepared as described by Dominguez Rubio et. al.^13^ with minor modifications. Briefly, strains *L. casei* BL23 and a mutant strain from *L. casei* BL23, which carries the plasmid pT1-GR::p127, encoding for both red fluorescent (RFP) and green fluorescent protein (GFP) under the control of a truncated form of the *prtP,prsA* promoter^59^ were grown in Man, Rogosa and Sharpe (MRS) medium (Difco) at 37 °C for 48 h under static conditions. Strain *L. casei* BL23 [pT1-GR::p127] was grown in the presence of 5 µg/ml of eritromycin. The culture was centrifuged at 4,000*g* for 25 min at 4 °C, the resulting supernatant was filtered using a 0.45 µm pore size and then centrifuged at 37,000 rpm (average 100,000*g*) for 2 h in a Beckman Coulter ultracentrifuge using a Type Ti70 rotor. Aliquots of the resulting supernatants were mixed with SDS-PAGE buffer for subsequent Western-Blot analysis and the EV pellet was washed once in phosphate-buffered saline (PBS, Biowest). A possible formation of EV aggregates after high-speed centrifugation was previously described^60^, hence to eliminate macroscopically visible aggregates the suspension was subjected to low-speed centrifugation at 8,000 g for 2 min. EVs were stored until their use at − 80 °C in small aliquots to prevent multiple freeze–thaw cycles. Before freezing, surface protein content was determined using the BCA method (Sigma-Aldrich). EVs were also prepared with polyethylene glycol (PEG 6000), a procedure describe to have an excellent yield of vesicles for any analytical purposes^35,61^. Briefly, the method consisted in the centrifugation of 2 l of *L. casei* BL23 culture at 10,000 g for 20 min at 4 °C in 250 ml bottles. Then the culture supernatant was filtered through a filtration unit with 0.22 µm pore size (Millipore Filtration Unit Stericup-GP) and mixed with equal volume of cold 16% PEG 6000 in 1 M NaCl; the mixture was kept at 4 °C overnight and centrifuged at 10,000*g* for 20 min at 4 °C. The pellet containing EVs was resuspended in 8% PEG, 0.5 M NaCl and centrifuged in Eppendorf tubes at 11,000 rpm. Pellets were then resuspended very gently in cold PBS and frozen at − 80 °C until use.

### Eukaryotic cell culture and phospho-EGFR assay

The human colon carcinoma cell line T84 was routinely maintained in DMEM/F-12 1:1, supplemented with 10% fetal bovine serum (Sigma-Aldrich), 10 mM Hepes, 100 µg/ml of penicillin G, 100 U/ml of streptomycin and 2 mM L-glutamine. The human colon tumorigenic cell line HT-29 was maintained in DMEM supplemented with 10% fetal bovine serum, 1 mM sodium pyruvate, 100 µg/ml of penicillin G, 100 U/ml of streptomycin and 2 mM L-glutamine. Both cell lines were cultured at 37 °C, 5% CO_2_ under a humidified atmosphere. In order to check the possible toxic effect of BL23 EVs on HT-29 cells, we used the resazurin colorimetric viability assay as described before^62^.

To test for activation of phospho-EGFR in T84 cells, cells were seeded at 320,000 cells/well in 24-wells plates and stimulated with EVs or purified His-tagged P40 and P75^28^ when cells reached about 80% of confluence. 16–20 h prior to stimulation, cells were serum-starved and culture medium was replaced with medium without FBS. Cells were stimulated with the indicated amounts of EVs for 30 or 60 min and purified proteins for 60 min, culture medium was aspirated and cells were harvested directly in SDS-PAGE loading buffer for immunoblotting. Positive control of stimulation was carried out with epidermal growth factor (EGF) at 30 ng/ml in the same conditions.

### Generation of a NF-κB reporter cell line

The plasmid pNiFty2-SEAP (Invivogen) encoding for a secreted form of the human embryonic alkaline phosphatase (SEAP) under the control of the NF-κB binding site was stably transfected into HT-29 cells using the X-treme GENE HP DNA Transfection Reagent (Roche) following the manufacturers’ instructions. After transfection, cells were maintained using 400 µg/ml of zeocin for about 3–4 weeks until clones were macroscopically visible. Then clones were subcultured, characterized and selected in response to the inflammatory stimulants TNF-α, LPS and *E. coli*. Once NF-κB responsive clones were selected, the stable reporter cell line was maintained routinely in 150 µg/ml of zeocin.

For analysis of NF-κB activation in response to EVs, cells were seeded at 60,000 cells/well in 96-well plates as described before^63^. Cells were grown 24 h before experiment and then stimulated with different EV concentrations in a final volume of 100 µl. After 24 h of stimulation, SEAP (secreted alkaline phosphatase) activity in the cell culture supernatant was quantified using p-nitrophenyl phosphate as phosphatase substrate according to the manufacturers’ instructions (Thermo Scientific, Ref.: 37,620). The yellow-coloured reaction products were detected using a microplate reader (Multiskan Ascent) at 414 nm.

### Transmission electron microscopy (TEM)

TEM as well as sample preparation was carried out at the microscopy facility, SCSIE, of the University of Valencia. Briefly, the purified EV samples were applied to copper grids and stained with 2% uranyl acetate. Samples were examined in a transmission electron microscope Jeol JEM-1010 (80 kV) with a AMT RX80 (8 Mpx) digital camera. TEM images were analysed and vesicle diameters were measured using the software NIS-Elements (Nikon) for frequency size distribution.

### Western-blot

Western-Blot analysis were carried out as previously described^28^. Briefly, 45 µg of protein from T84 samples were separated on 8% (for detection of p-EGFR, p-Akt, p-ERK 1/2 and β-actin) and 10% (P40 and P75) SDS-PAGE gels and electrotransferred to Hybond-ECL membranes (GE Healthcare). Membranes were blocked in a 5% (w/v) non-fat dry milk solution in 50 mM Tris–HCl pH 7.6, 150 mM NaCl (TBS) containing 0.1% (v/v) Tween-20. Rabbit polyclonal anti-P40N, anti-P75N^28^, mouse—monoclonal anti-β-actin (Ref. A-2228, Sigma-Aldrich) were diluted 1:5.000 in 5% (w/v) non-fat dry milk in TBS-T and incubated for 2 h, rabbit polyclonal antibodies anti-p-EGFR (Tyr1068, #2234, Cell Signaling), anti-p-Akt (Ser473, #9271, Cell Signaling) and anti-p-Erk1/2 (Thr202/Tyr204, #9101, Cell Signaling) were incubated O/N at 4 °C in 5% BSA in TBS-T. Blots were incubated with secondary goat anti-rabbit and anti-mouse (β-actin) HRP-conjugated antibodies and signals were detected using the ECL advance chemiluminescent reagents as described by the supplier (GE Healthcare).

Western blotting was also implemented for the detection of *L. casei* BL23 LTA following a previously described procedure^64^, briefly, it required SDS free loading buffer and electrophoresis in 17.5% polyacrylamide gels (SDS free). Anti-*Staphylococcus aureus* LTA mouse monoclonal antibody (G43J) (Invitrogen) was used as primary antibody, and the rest of the procedure was followed as above. Detection of LTA required to use larger amounts of BL23 *EVs* than for the detection of P40 and P75, hence EVs were prepared with the PEG6000 procedure, so that a minimum of EVs equivalent to 2.85 μg protein had to be loaded in the gel to detect LTA (Fig. S6).

### Proteinase K (PK) assay

PK assay was performed as described in^34^. Briefly, purified *L. casei* BL23 EVs (1.5 µg) either intact or lysed for 2 h at 37 °C with 0.1 M EDTA were treated with proteinase K (Roche) at 100 µg/ml for 30 min. Reactions were stopped by adding SDS-PAGE loading buffer and aliquots were immunoblotted with anti-P40N and anti-P75N.

### Light scattering determination of vesicles’ size

The average size of BL23 EV was determined by DLS using a Zetasizer NanoZS (Malvern Panalytical Ltd, U.K.) with similar settings as those reported by others^12^. Before the analysis, samples were filtered through 0.20 µm microbiological filters (Filtropur S, Sarstedt AG & Co KG, Germany). Measurement temperature was 20 °C in disposable polystyrene cells of small size, using 400 µl samples that were stabilized during 60 s previous to the analysis. Samples were measured by triplicate, with 8 cycles of 8 s each measurement, followed by a confirmative determination by accumulation of 10 cycles of 20 s. The refraction index of the samples was fixed to 1.330 and the viscosity to 1.0031 mPa/s. The equipment software calculated the average particle size by third order fitting autocorrelation function as described by the manufacturer (Malvern Panalytical Ltd, U.K.).

### Surface plasmon resonance (SPR) analysis

Binding of LTA to P40 and P75 was determined by SPR using a Biacore T100 instrument (Biacore, GE Healthcare). *Staphylococcus aureus* lipoteichoic acid (LTA) were purchased from SIGMA, and purified P40 and P75 were immobilized on the surface of CM5 chips (GE Healthcare). Immobilization was achieved with the Amine Coupling Kit (GE Healthcare) following the manufacturer’s instructions. Each one of the proteins were immobilized at 4400–5900 resonance units (RU). P40 and P75 were immobilized in channels 2 and 4, respectively, of the CM5 chips and the channels 1 and 3 were left as references. Several fold dilutions of LTA (2,5–80 µM) and *L. casei* BL23 EVs (1 and 32 μg /ml of EVs total protein), including repetitions and blanks, were injected in the CM5 chip at a flow rate of 30 μl/min in 100 mM phosphate buffer pH 5.2 at 25 °C. Kinetic parameters were determined by the Single Cycle procedure at a flow rate of 10 µl/min and constant evaluations obtained with the Biacore Evaluation Software.

## Supplementary information

Supplementary Information
