# Supplementary material for: Lactobacillus casei extracellular vesicles stimulate EGFR pathway likely due to the presence of proteins P40 and P75 bound to their surface
Source: Sci Rep. 2020 Nov 6;10:19237. doi: 10.1038/s41598-020-75930-9 (PMC7648624; doi:10.1038/s41598-020-75930-9)
Supplement: Supplementary file 1 — Supplementary Information [file 41598_2020_75930_MOESM1_ESM.pdf]

Supplementary Information

Lactobacillus casei Extracellular Vesicles Stimulate EGFR Pathway Likely Due to the Presence of Proteins P40 and P75 Bound to their Surface

Christine Bäuerl [1], José M. Coll-Marqués[1], Carmen Tarazona-González[1] and Gaspar Pérez-Martínez[1*]

[1] *Laboratory of Lactic Acid Bacteria and Probiotics, Department of Food Biotechnology, Instituto de Agroquímica y Tecnología de Alimentos, Consejo Superior de Investigaciones Científicas (CSIC) (Spanish National Research Council), Avenida Agustín Escardino, 7, 46980 - Paterna, Valencia, Spain*

[*] Corresponding author: Gaspar Pérez-Martínez gaspar.perez@iata.csic.es

(A) Kinetic model of 1:1 binding

	Curve	ka (1/Ms)	kd (1/s)	KD (M)	Rmax (RU)	Conc (M)	tc	Flow (ul/min)	kt (RU/Ms)	RI (RU)	Chi^2 (RU^2)	U-value
**P75**		1,66E+07	0.06319	3,80E-03	2,25E+07		3,59E+12				418	2
	Cycle: 5					2,50E-06		10.00	7,74E+12	33.51		
						5,00E-06				51.31		
						1,00E-05				85.29		
						2,00E-05				20.94		
						4,00E-05				41.98		
**P40**		1,71E+13	74.00	4,34E-06	104.1		1,23E+10				502	12
	Cycle: 5					2,50E-06		10.00	2,65E+10	47.18		
						5,00E-06				98.45		
						1,00E-05				164.0		
						2,00E-05				148.7		
						4,00E-05				242.6		

(B) Kinetic model with heterogeneous ligand

	Curve	ka1 (1/Ms)	kd1 (1/s)	KD 1 (M)	ka2 (1/Ms)	kd2 (1/s)	KD2 (M)	Rmax1 (RU)	Rmax2 (RU)	Conc (M)	tc	Flow (ul/min)	kt (RU/Ms)	RI (RU)	Chi^2 (RU^2)	U-value
**P75**		1,10E+08	0.1719	1,56E-03	3,54E+07	0.04637	1,31E-03	4764	4708		1,90E+12				410	N/A
	Cycle: 5									2,50E-06		10.00	4,09E+12	31.77		
										5,00E-06				47.82		
										1,00E-05				78.42		
										2,00E-05				7.799		
										4,00E-05				17.50		
**P40**		5,99E+09	0.03150	5,26E-06	1,08E+08	0.007651	7,06E-05	104.9	0.8752		1,07E+10				507	N/A
	Cycle: 5									2,50E-06		10.00	2,31E+10	48.01		
										5,00E-06				101.4		
										1,00E-05				170.1		
										2,00E-05				155.5		
										4,00E-05				244.2		

TABLE S1.- Kinetic constants calculated for LTA as analyte on P40 and P75 at 25°C and their adjustment to 1:1 and heterogeneous ligand binding models.

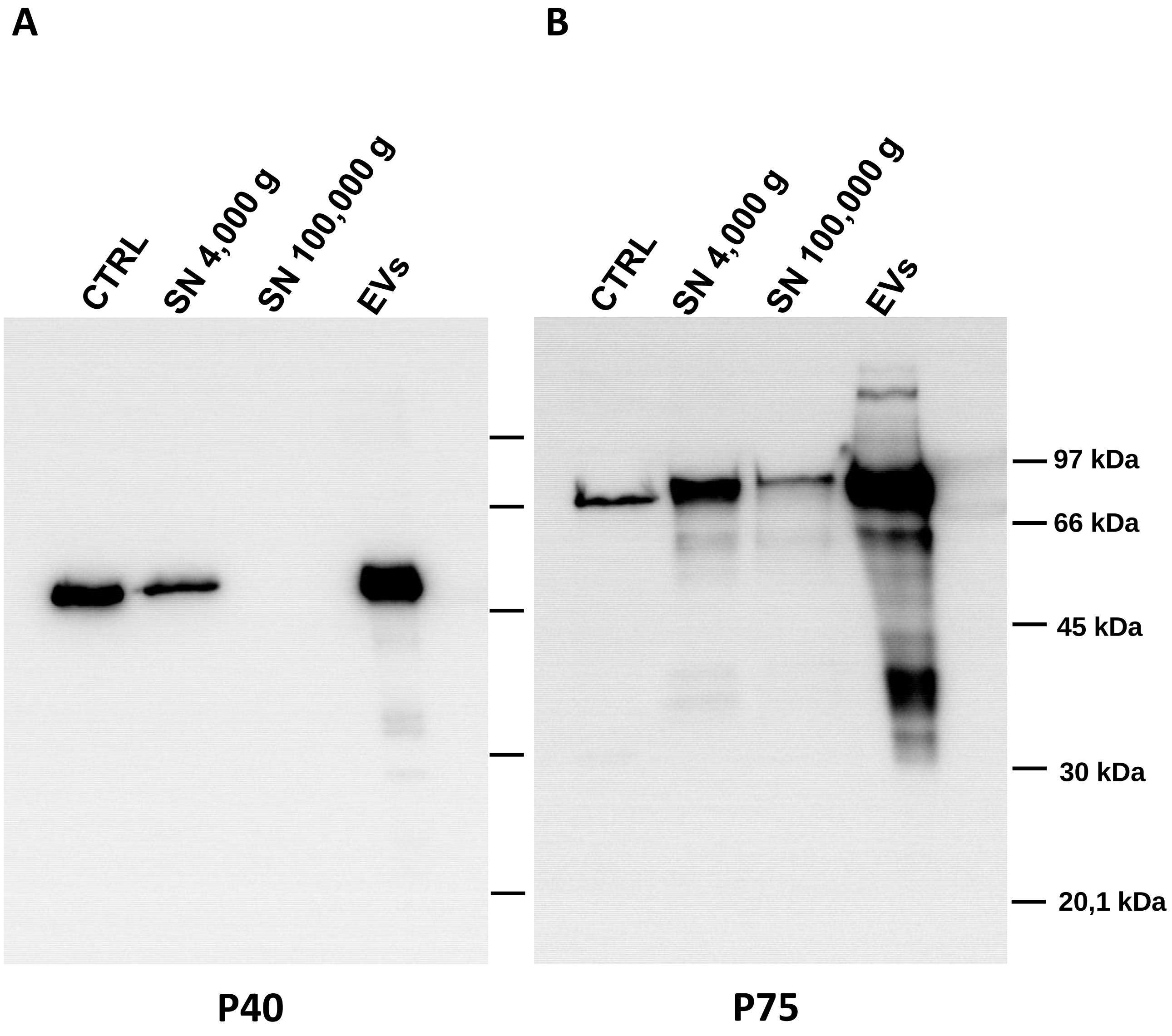

Figure S1. Full length Western-Blot for (A) P40 and (B) P75 protein expression shown in **Figure 2A** in the main manuscript using a 5-s- exposure time for image aquisition.

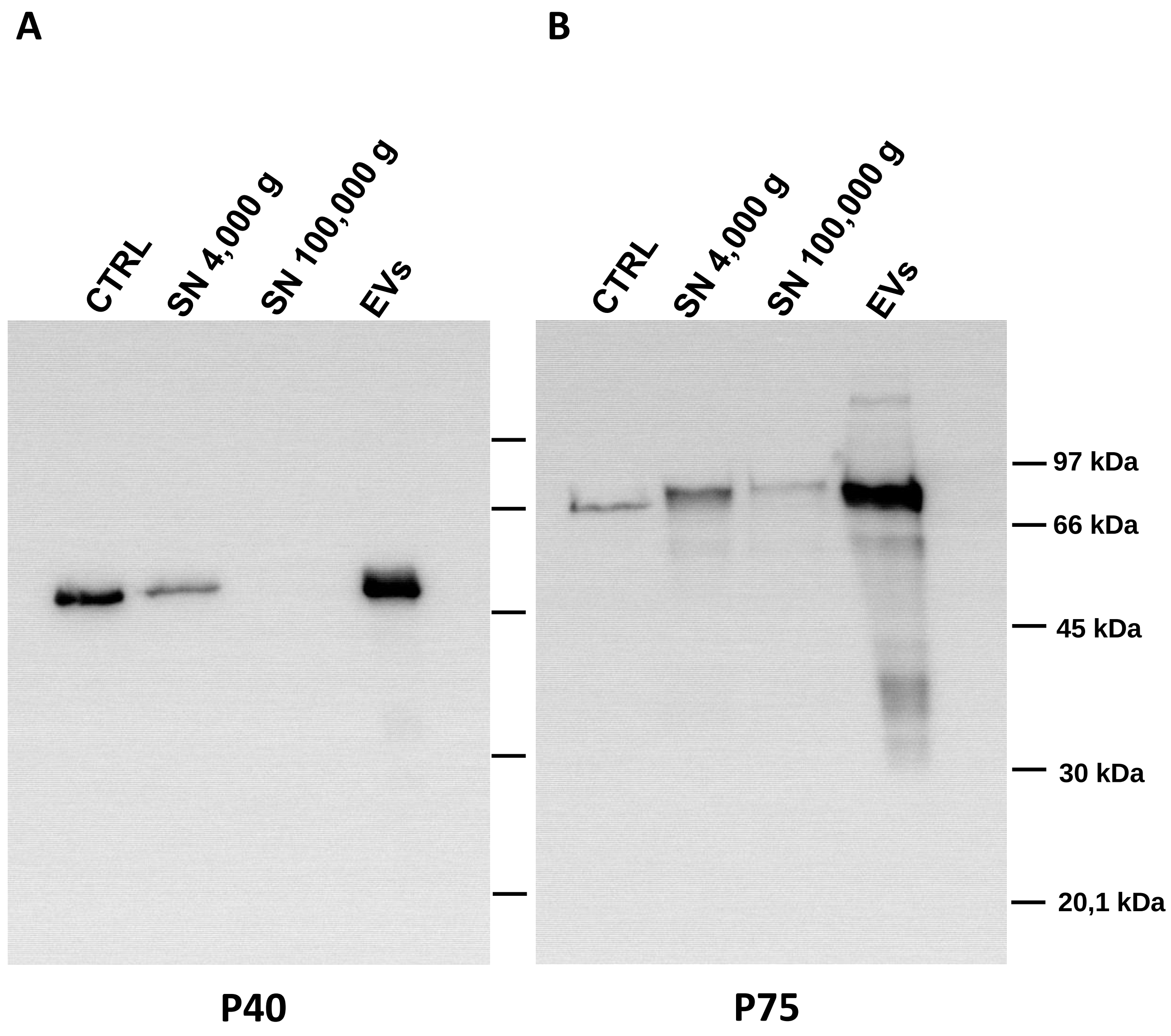

Figure S2. Full length Western-Blot for (A) P40 and (B) P75 protein expression shown in **Figure 2A** in the main manuscript using a 1-s exposure time for image aquisition.

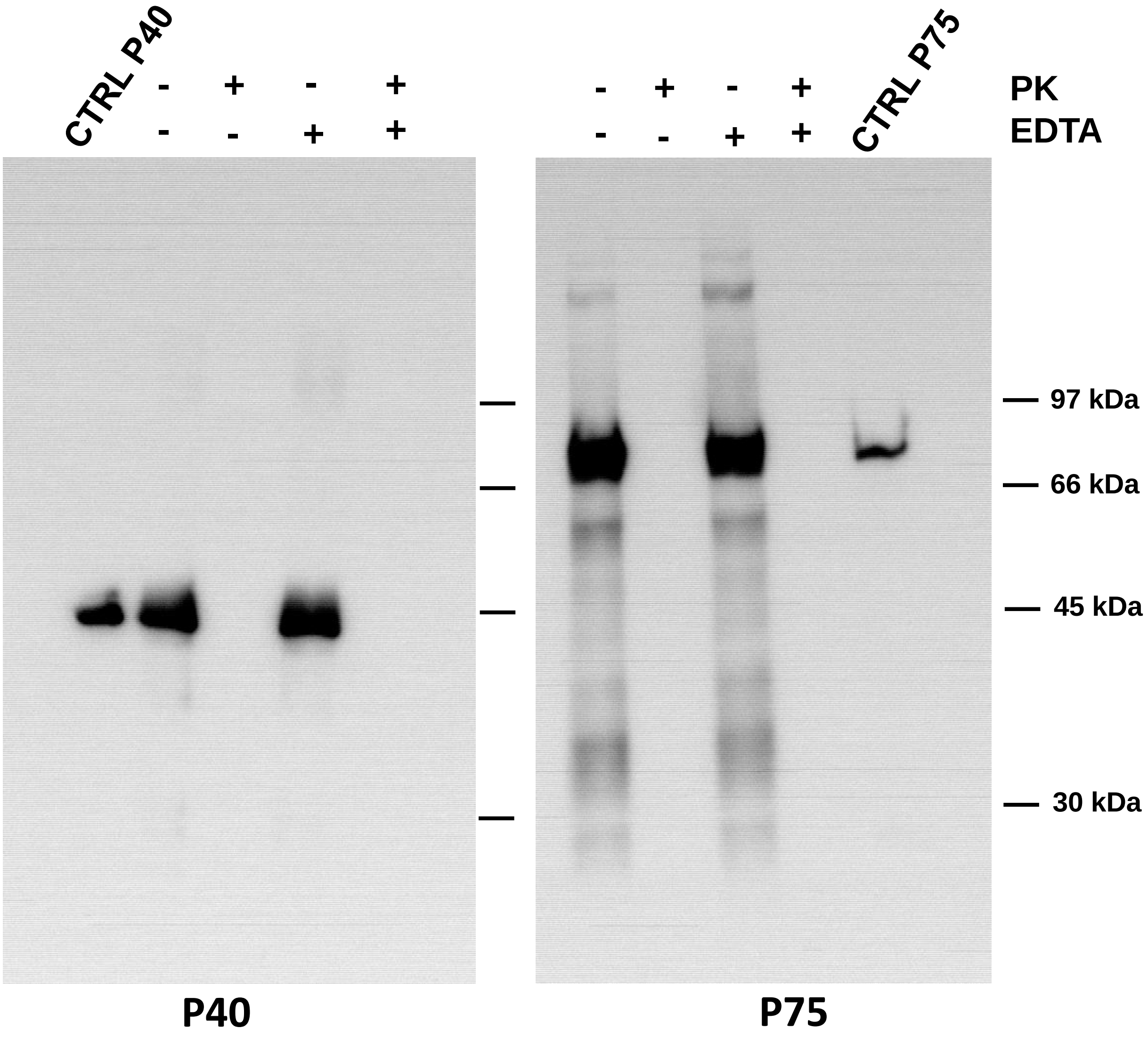

Figure S3. Full length Western-Blot for (A) P40 and (B) P75 protein expression shown in **Figure 2B** in the main manuscript using a 2-s exposure time for image aquisition. (CTRL) control lanes contain 10 ng of purified recombinant His-tagged P40 or P75.

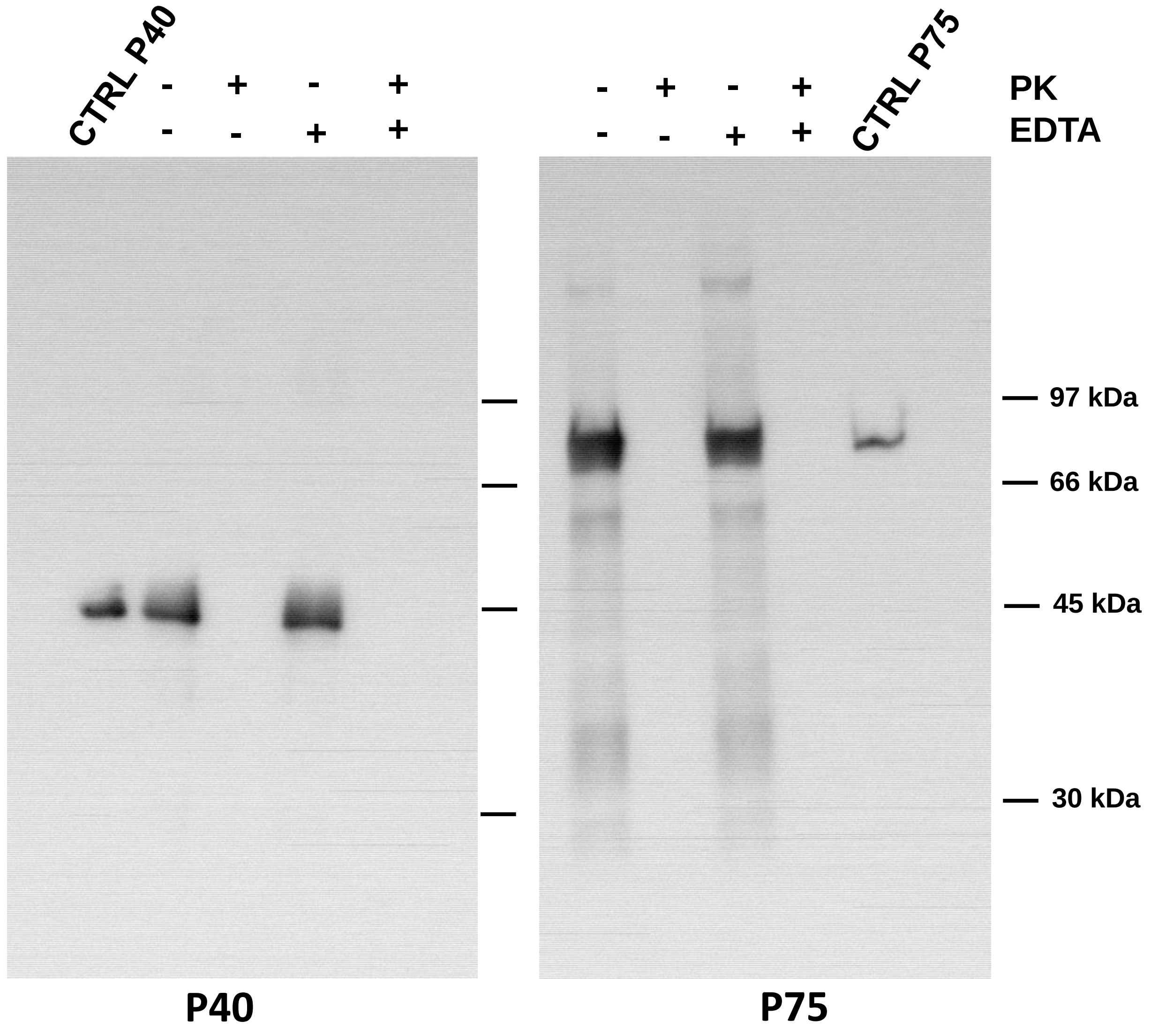

Figure S4. Full length Western-Blot for (A) P40 and (B) P75 protein expression shown in **Figure 2B** in the main manuscript using a 1-s exposure time for image aquisition. (CTRL) control lanes contain 10 ng of purified recombinant His-tagged P40 or P75.

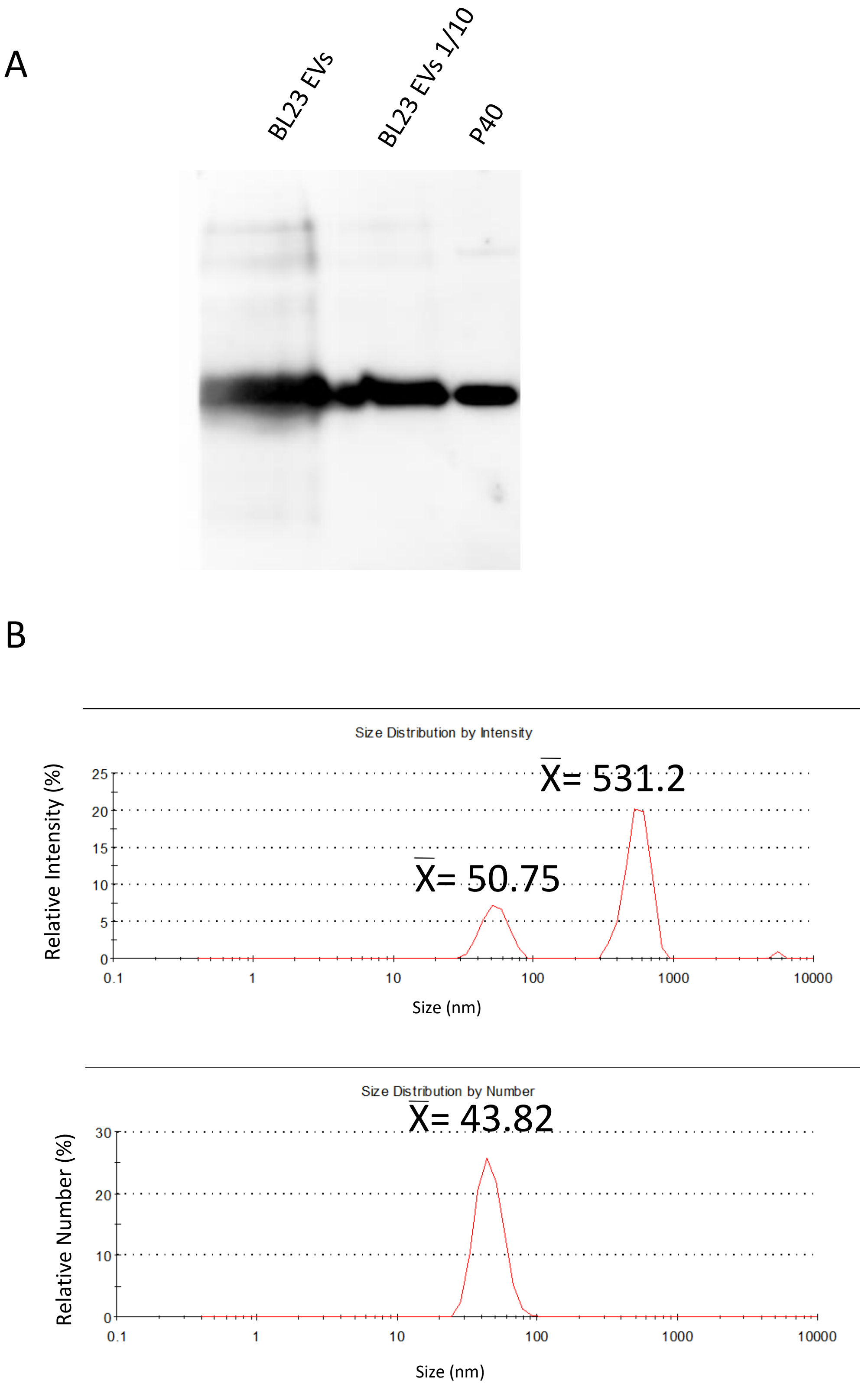

Figure S5.- Features of the *L. casei* EVs obtained by the PEG6000 procedure. (A) Western blot detecting P40 in a 12% SDS-PAGE. Tracks from left to right were loaded with BL23 EVs (2.85 µg protein), BL23 EVs (0.28 µg protein), purified P40. (B) DLS analysis of vesicle sizes showed a small size of vesicles, similar to those obtained by ultracentrifugation, but they had slightly larger size: Size *vs.* Intensity 50.75 nm and Size *vs.* Number 43.82 nm; however sedimenting aggregates yielded low quality analysis of some samples and had to be removed by filtration through filters of 0.22 µm pore size.

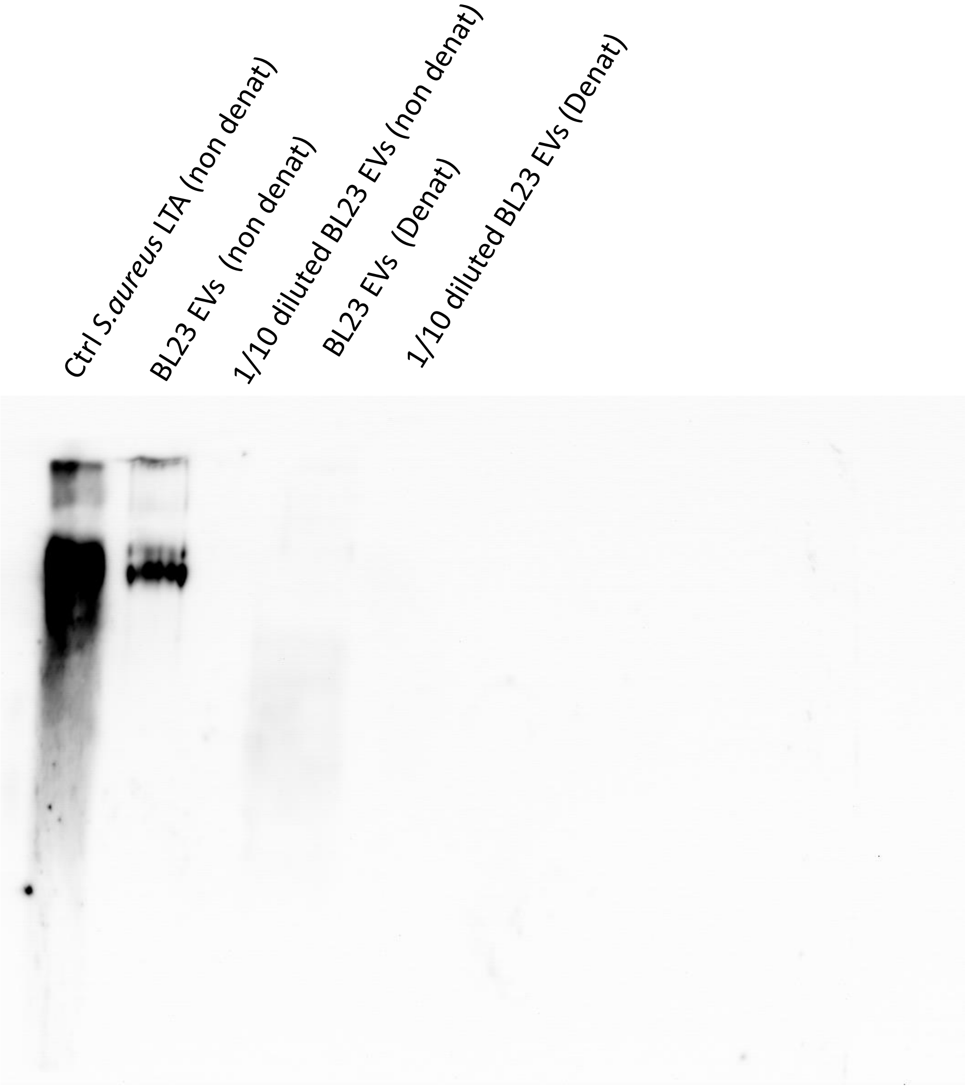

Figure S6.- Full length Western-Blot for LTA on EVs from a non-denaturing 17.5% PAGE loaded with LTA (Control) and *L.casei* BL23 Evs as shown in **Figure 3A** in the main manuscript.
Lanes contained samples as indicated. The annotation in parenthesis (non denat) indicates that samples were loaded using non-denaturing loading buffer without heating, while (Denat) indicates that samples were heated in denaturing buffer previous to loading the gel. The rest of the gel contained other samples not relevant fot this work.

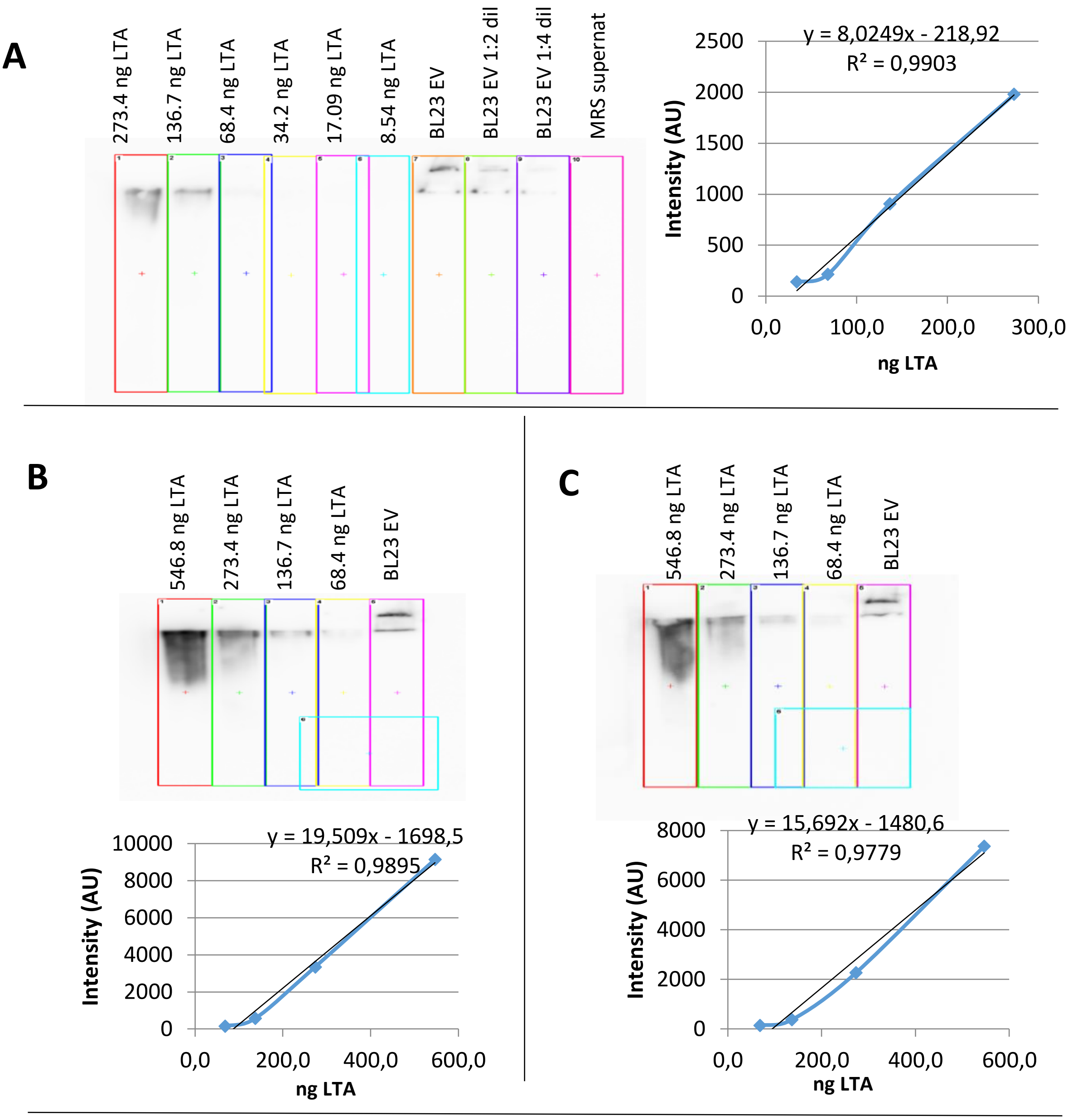

QUANTIFICATION OF LTA IN *L.casei* BL23 EV

	ng LTA/ µl sample	ng LTA / µg EV protein*
Western A	16.669	61.966
Western B	14.865	55.260
Western C	16.009	59.513
**AVERAGE(St.Dev.)**	**15.848 (0,913)**	**58.913 (3.393)**

(*) Calculated protein content of *L. casei* BL23 EV samples was 269 µg protein/ml

Figure S7.- Quantification of LTA in *L. casei* BL23 EVs by western blot using mouse anti-*S. aureus* LTA as primary antibody. Panels A, B and C show different replica assays. A wider range of concentrations was tested in Panel A but only the four with higher concentration were used for the regression. Also three dilutions of the samples and a sample of the culture supernatant (MRS) were also loaded.

Western blots were performed as described in Materials and Methods. Acurate densitometry of complete western blot lines was obtaind with an Amersham Imager 680 (Cytiva Europe GmbH, Germany) and total intensity of lines in the blots were quantified with NIS-Elements (Nikon, Japan).

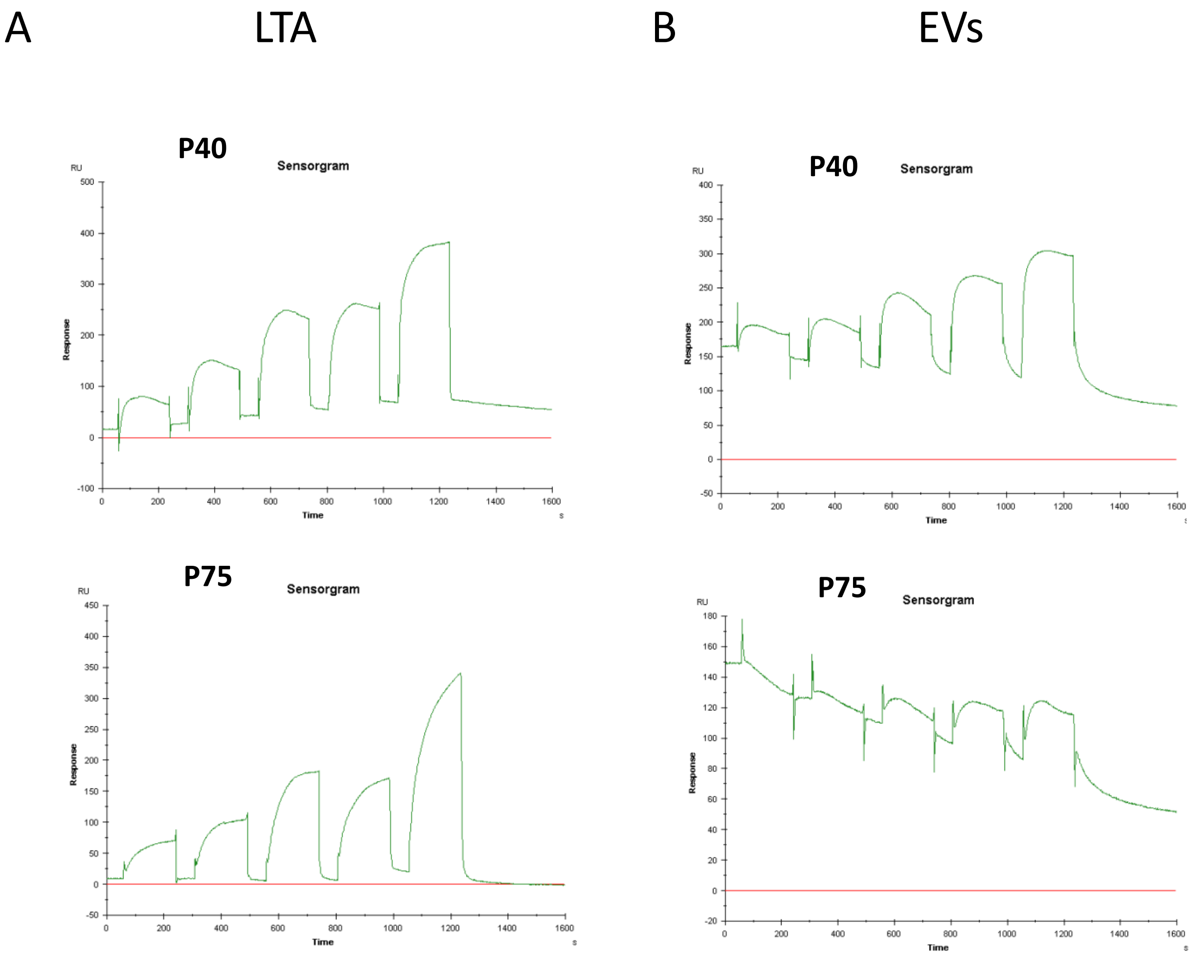

Figure S8.- SPR sensorgrams of Single Cycle kinetics assays where a series of LTA and EVs (analyte) concentrations were run in one cycle, without regeneration between sample injections. Binding assays of LTA (A) and BL23 EVs (B) to P40 and P75.

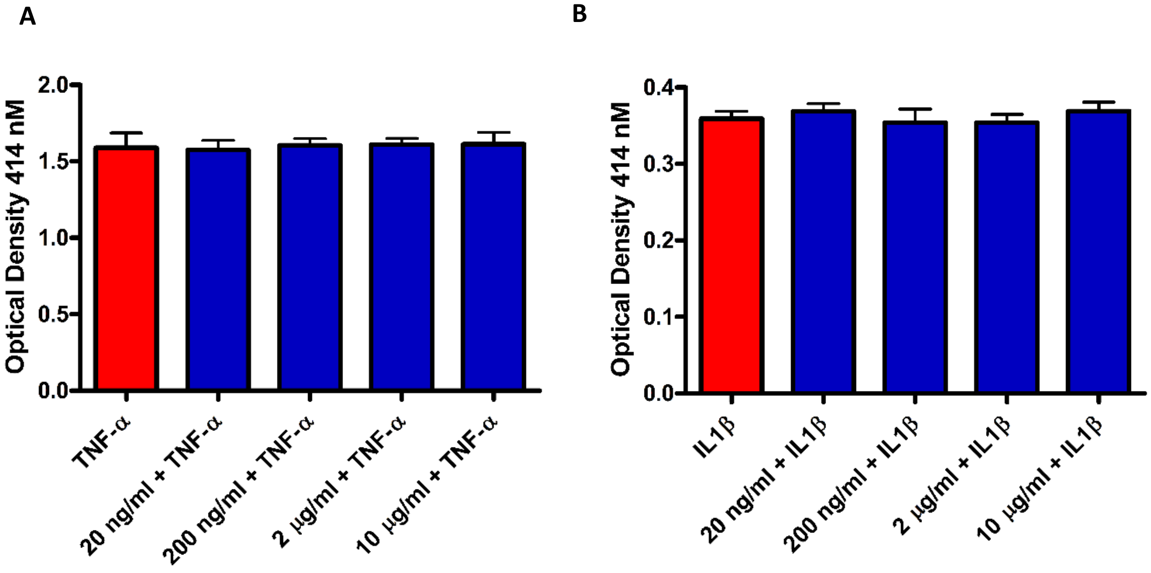

Figure S9- Assay of the anti-inflammatory activity of EVs using transfected HT29 intestinal epitelial cells [pNiFty2-SEAP] by detection of the secreted alkaline phosphatase and exposed to different EVs concentrations given as total absorbance units. Barr Graphs in show the (absence of) anti-inflammatory effect of *L.* casei BL23 vesicles after induction with TNFα (A) and IL1β (B).

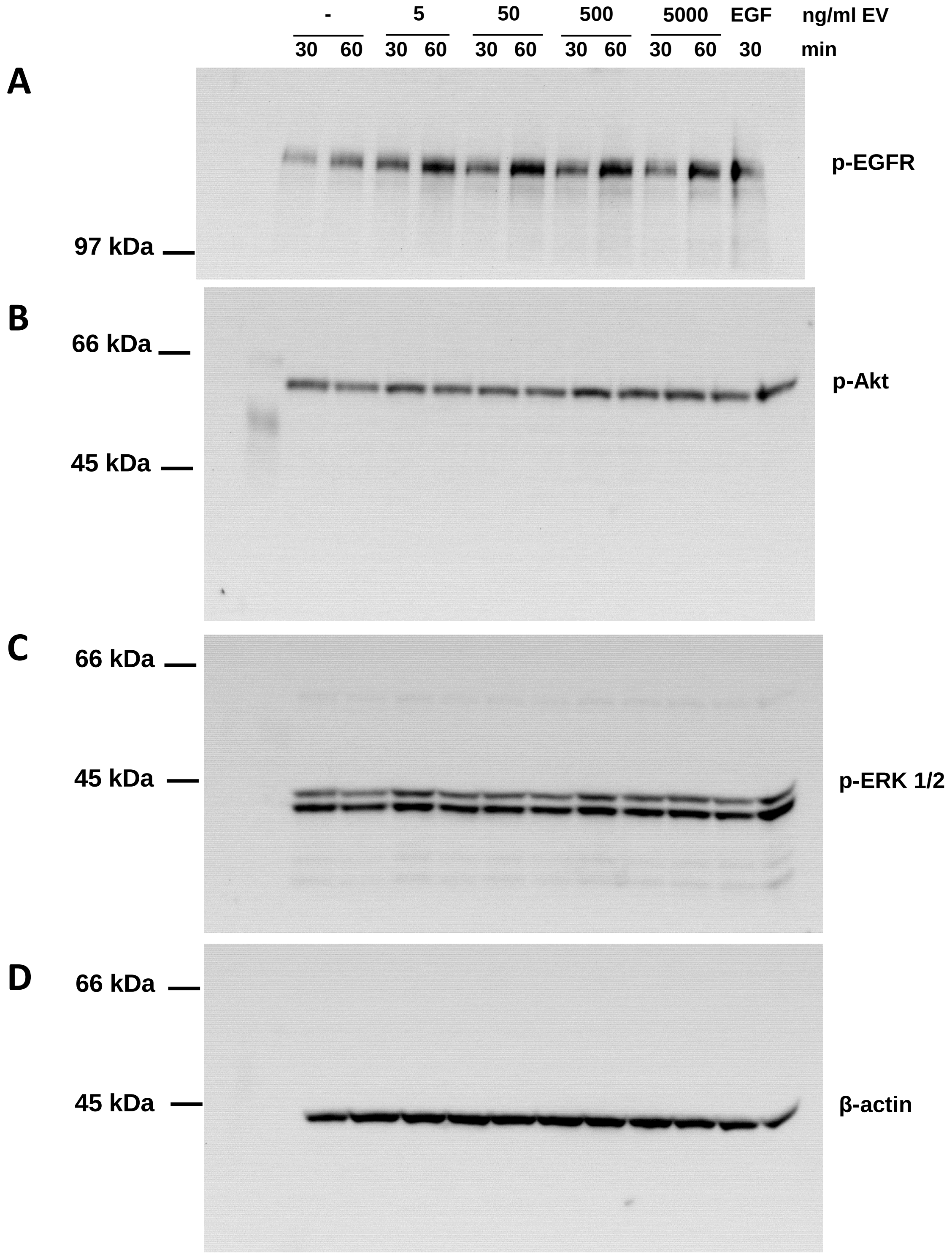

Figure S10.- Full length Western-Blot for (A) p-EGFR (B) p-Akt (C) p-ERK1/2 and (D) β-actin protein expression shown in composed **Figure 5A** in the main manuscript.

P40

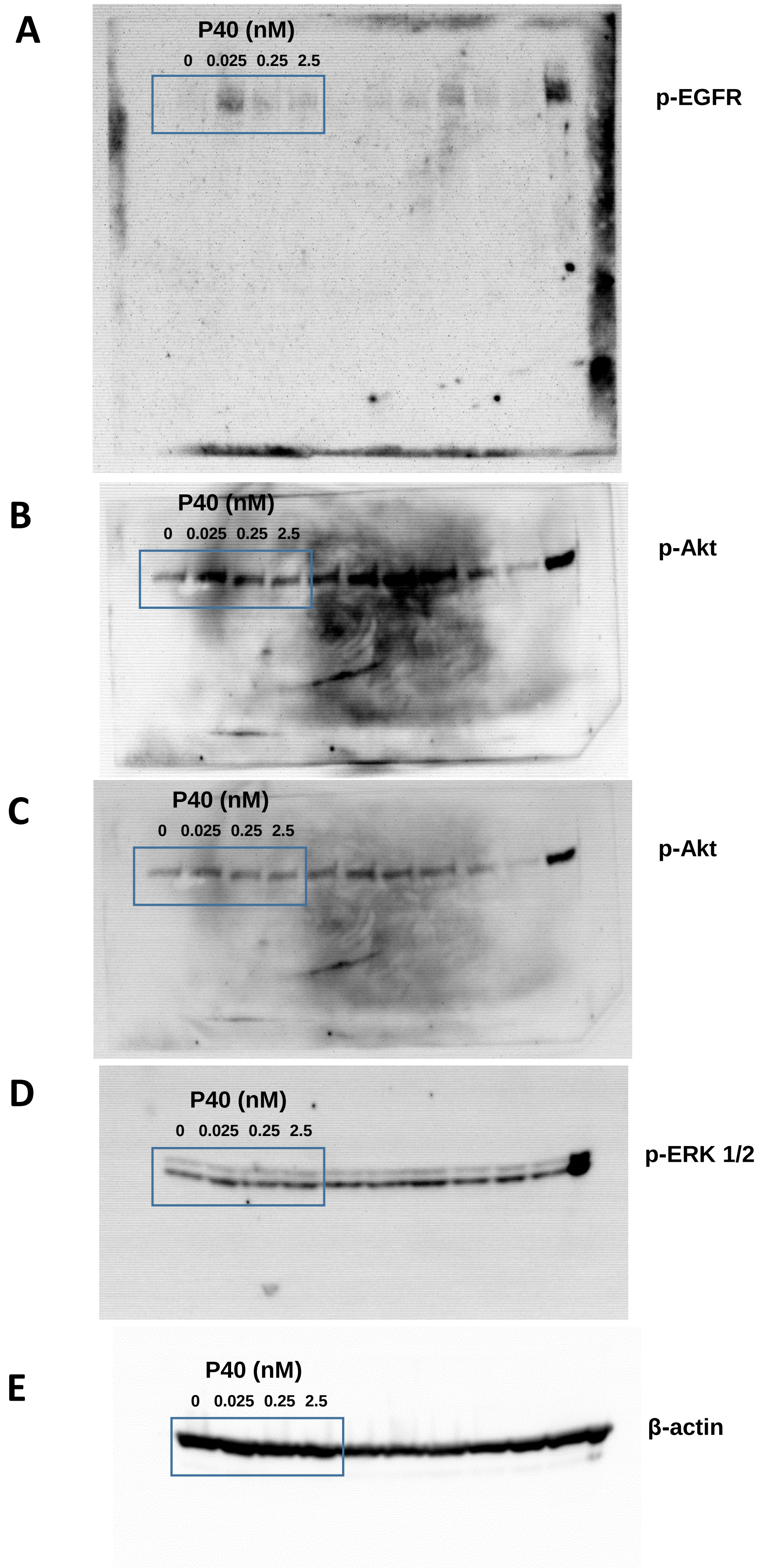

Figure S11.- Full length Western-Blot for (A) p-EGFR (B) p-Akt using 5 min of exposure time (C) p-Akt using 3 min of exposure time (D) p-ERK1/2 and (E) β-actin protein expression in response to purified P40 in T84 IEC and shown in composed **Figure 5C** in the main manuscript. Note that the rectangle boxes indicate the areas that were cropped out to make the composite image in the main figure.

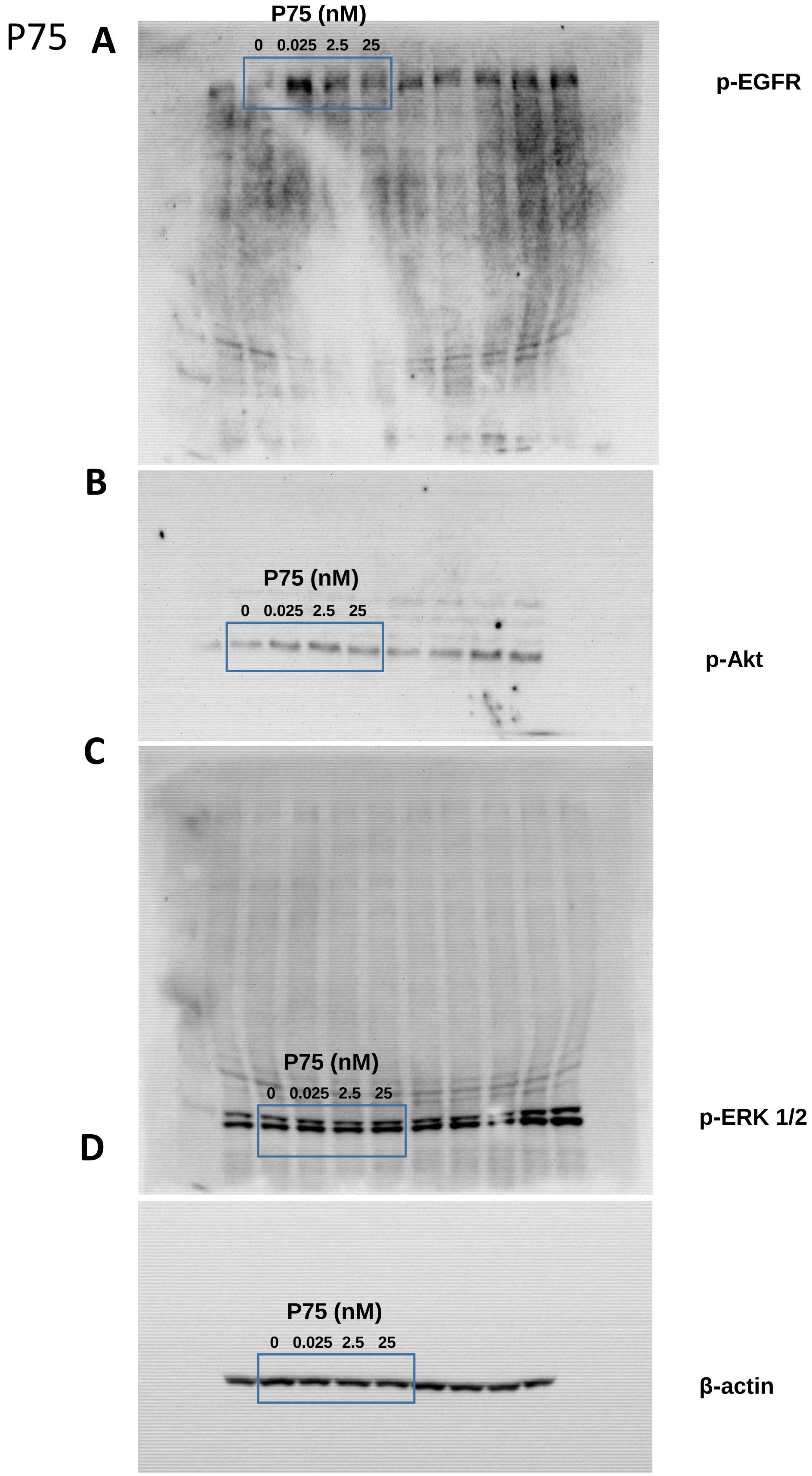

Figure S12.- Full length Western-Blot for (A) p-EGFR (B) p-Akt (C) p-ERK1/2 and (D) β-actin protein expression in response to purified P75 in T84 IEC and shown in composed **Figure 5C** in the main manuscript. Note that the rectangle boxes indicate the areas that were cropped out to make the composite image in the main figure. Images shown for (A) p-EGFR and (C) p-ERK1/2 are from the same blot, whereas images from (B) p-Akt and (D) β-actin correspond to another blot out of the three replicates.
